# Functional Outcomes After Decompressive Surgery in Patients with Malignant Space-Occupying Cerebellar Infarction

**DOI:** 10.3390/neurolint16060094

**Published:** 2024-10-28

**Authors:** Enayatullah Baki, Lea Baumgart, Victoria Kehl, Felix Hess, Andreas Wolfgang Wolff, Arthur Wagner, Moritz Roman Hernandez Petzsche, Tobias Boeckh-Behrens, Bernhard Hemmer, Bernhard Meyer, Jens Gempt, Silke Wunderlich

**Affiliations:** 1 Department of Neurology, Klinikum Rechts der Isar, School of Medicine and Health, Technical University of Munich, 81675 Munich, Germany; 2 Department of Neurosurgery, University Medical Center Hamburg-Eppendorf, 20251 Hamburg, Germany; 3 Institute of AI and Informatics in Medicine, Klinikum Rechts der Isar, School of Medicine and Health, Technical University of Munich, 81675 Munich, Germany; 4 Department of Neurosurgery, Klinikum Rechts der Isar, School of Medicine and Health, Technical University of Munich, 81675 Munich, Germany; 5 Department of Diagnostic and Interventional Neuroradiology, Klinikum Rechts der Isar, School of Medicine and Health, Technical University of Munich, 81675 Munich, Germany; 6 Munich Cluster for Systems Neurology (SyNergy), 81377 Munich, Germany

**Keywords:** ischemic stroke, cerebellar infarction, decompressive surgery

## Abstract

**Background and Purpose**: Decompressive surgery is a potentially life-saving treatment in patients with malignant space-occupying cerebellar infarction. However, there is only limited literature on functional outcomes and complications after surgery. Our aim was to establish markers which predict poor outcome. **Methods**: We retrospectively analyzed data of all patients who underwent surgery due to malignant swelling of a space-occupying cerebellar infarction in our hospital between 2005 and 2023. Statistical analyses were conducted on multiple parameters to identify predictors of poor functional outcome (mRS 4–6) 90 days after surgery. Complications during hospitalization were reviewed for each patient. **Results**: In total, 58 patients received decompressive surgery. The 90-day mortality rate was 27.6% (n = 16). A good functional outcome (mRS 0–3) 90 days after surgery was achieved in 24 patients (41.4%). Multivariable analysis revealed multiple factors associated with a poor outcome on day 90 (mRS 4–6): a higher premorbid mRS score (OR 2.715 [95% CI, 1.166–6.323]; *p* = 0.021), higher NIHSS score on admission (OR 1.088 [95% CI, 1.014, 1.168]; *p* = 0.019) and the presence of an additional brainstem infarction (OR 7.035, [95% CI, 1.255, 39.424], *p* = 0.027). Hyperactive delirium was associated with good clinical outcome (OR 0.020 [95%CI, 0.001–0.623]; *p* = 0.026). Aspiration pneumonia (n = 22, 37.9%), urinary tract infection (n = 15, 25.9%), and hyperactive delirium (n = 8, 13.8%) were the most common complications during hospitalization. **Conclusions**: Decompressive surgery is a safe, life-saving treatment for malignant space-occupying cerebellar infarction. Higher premorbid mRS, higher NIHSS score on admission and the presence of brainstem infarction are associated with a poor functional outcome.

## 1. Introduction

Malignant swelling can occur as a severe complication in space-occupying cerebellar infarction. The compression of the brainstem and the obstruction of the flow of cerebral spinal fluid will become fatal if left untreated [1]. Despite decompressive surgery of the posterior fossa being the only acute life-saving treatment option, there is only a little evidence regarding the clinical benefits. The aim of this study was to analyze the functional outcomes and complications after decompressive surgery in patients with malignant space-occupying cerebellar infarction and to identify predictors of a poor functional outcome.

## 2. Methods

The study was conducted in accordance with the guidelines of the Declaration of Helsinki. The data that support the findings of this study are available from the corresponding author upon reasonable request.

### 2.1. Data Sources and Patient Selection

Patients who were admitted to our hospital and underwent decompressive surgery for malignant swelling of space-occupying cerebellar infarctions between 2005 and 2023 were eligible for inclusion in this retrospective study. Malignant swelling was defined as a mass effect in the posterior cranial fossa, accompanied by a decrease in consciousness due to the compression of the brainstem and/or the development of obstructive hydrocephalus. Other potential underlying causes of decreased consciousness were ruled out [2,3].

We retrospectively identified patients who fulfilled the predefined criteria from our stroke quality assurance registry (Bavarian Working Group for Quality Assessment, BAQ).

### 2.2. Baseline Demographic, Clinical and Radiological Characteristics

Patients’ demographic and clinical characteristics included age, sex, premorbid functional status, vascular risk factors, modified Rankin Scale (mRS) and National Institutes of Health Stroke Scale (NIHSS) on admission and stroke subtypes according to the Trial of Org 10172 in Acute Stroke Treatment (TOAST) criteria.

Radiologic parameters, such as infarct volume, brainstem compression, hydrocephalus, side of infarction, vascular territory, basilar artery occlusion and the presence of additional brainstem infarction, were analyzed by experienced neuroradiologists. Infarct volume was measured using the well-known “ABC/2” method [4], in which A is the largest diameter of the selected slice by eye with the largest infarct area, B is the largest diameter perpendicular to the line above and C is the total number of slices of infarct seen multiplied by the slice thickness. The infarct volume was determined on the last cranial CT or MRI conducted before surgery.

Decompressive surgery was further analyzed with regard to the duration of the surgical procedure and the time span between symptom onset, respectively, malignant swelling and surgery. Other therapeutic interventions considered were the insertion of an external ventricular drain (EVD), intravenous thrombolysis with tissue plasminogen activator (tPA, alteplase) and endovascular treatment (EVT) of a basilar artery occlusion.

### 2.3. Endpoints

The primary endpoints were mortality and functional outcome, measured using the modified Rankin Scale (mRS) 90 days after surgery. Good functional outcome was defined as a score of 0–3 on the modified Rankin Scale and poor functional outcome as a score ≥4. To identify predictors of a poor functional outcome, the total cohort was divided into two groups: patients with good outcome and those with a poor outcome at day 90. Complications during hospitalization were evaluated as secondary endpoints for each patient individually.

### 2.4. Statistical Analysis

All statistical analyses were conducted using IBM SPSS Statistics for Windows (version 28.0.1.1). Descriptive statistics were presented as frequencies with percentages for categorical variables, as means with standard deviations for approximately normally distributed continuous variables and as medians with ranges for ordinal or not normally distributed variables, as appropriate. To determine potential predictors of poor functional outcome, we used univariate and multivariable logistic models and presented the results using the odds ratio (OR), 95% confidence interval (CI) and the *p*-value of the Wald statistic. Cumulative survival was analyzed using the Kaplan–Meier method. All statistical tests were performed on two sides at the global 5% significance level. Bonferroni–Holm correction was used to adjust the *p*-values for multiple testing.

## 3. Results

Our database included 58 patients who received decompressive surgery as an acute treatment for malignant space-occupying cerebellar infarction.

### 3.1. Survival

**Figure 1:**
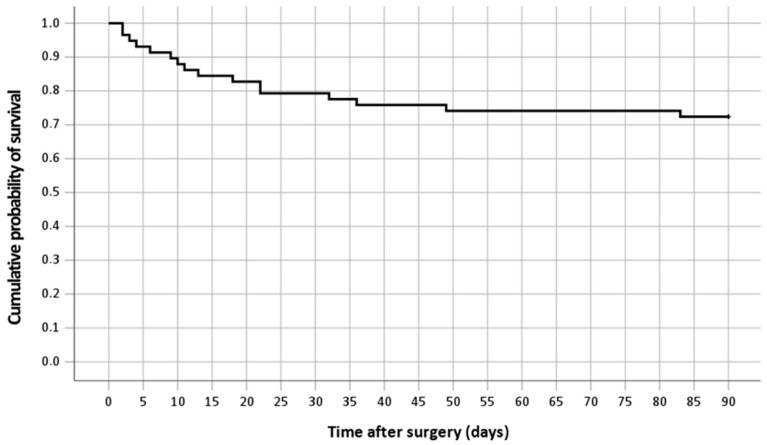
Kaplan–Meier curve showing the cumulative probability for survival in all patients.

Overall, 16 out of 58 patients died within 90 days after surgery, resulting in a 90-day mortality rate of 27.6%. Kaplan–Meier analysis, as illustrated in Figure 1, was used to model the cumulative probability for survival.

### 3.2. Functional Outcome

**Figure 2:**
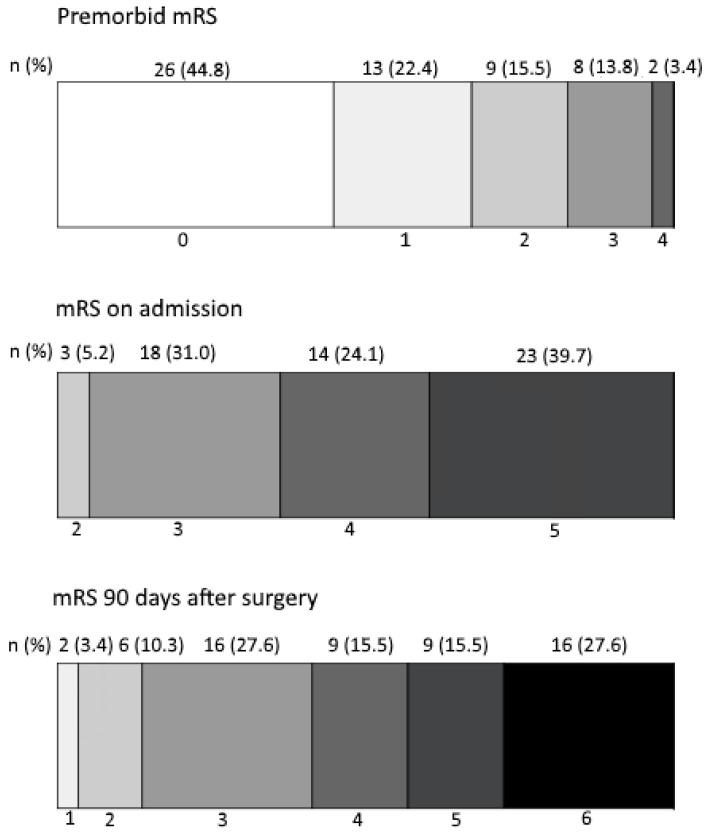
Distribution of mRS at premorbid, on admission and 90 days after surgery.

Overall, 34 out of 58 patients (58.6%) had a poor outcome at 90 days following decompressive surgery. Accordingly, 24 patients (41.4%) had a good outcome. The detailed distribution of mRS scores (premorbid, on admission and 90 days after surgery) is depicted in Figure 2.

### 3.3. Complications

Aspiration pneumonia was the most frequent complication during hospitalization (n = 22, 37.9%), followed by urinary tract infection (n = 15, 25.9%), hyperactive delirium (n = 8, 13.8%), cardiac arrhythmia (n = 7, 12.1%) and ventriculitis (n = 4, 6.9%).

**Figure 3:**
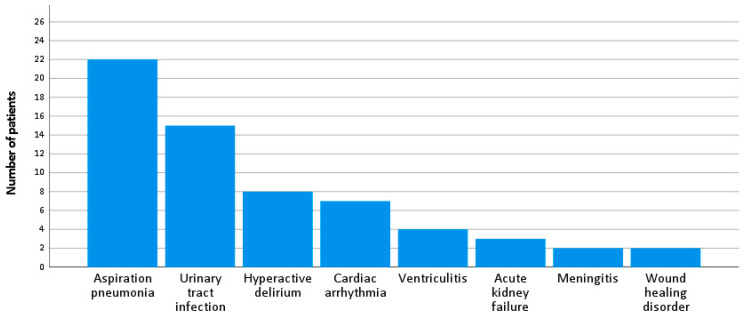
Complications during hospitalization.

Three patients (5.2%) suffered acute kidney failure and two patients (3.4%) developed meningitis after surgical treatment. Postoperative wound healing disorder with surgical site infection and leak of cerebrospinal fluid was reported in two patients (3.4%). All complications are depicted in Figure 3.

### 3.4. Comparison of Patients with Good and Poor Functional Outcomes

Comparison of demographic, clinical and radiological characteristics are summarized in Table 1. The same table also gives details on the univariate logistic regression analysis.

The multivariable logistic regression model, shown in Table 2, confirmed multiple variables associated with a poor functional outcome, as follows.

Among clinical characteristics, a higher premorbid mRS score (OR 2.715 [95% CI, 1.166–6.323]; *p* = 0.021) and a higher NIHSS score on admission (OR 1.088 [95% CI, 1.014, 1.168]; *p* = 0.019) were associated with a poor functional outcome.

Among imaging parameters, the presence of an additional brainstem infarction was also associated with a poor functional outcome (OR 7.035, [95% CI, 1.255, 39.424], *p* = 0.027).

After analyzing the most common complications as potential predictors of poor outcome, the occurrence of postoperative hyperactive delirium was associated with a good functional outcome (OR 0.020 [95%CI, 0.001–0.623]; *p* = 0.026).

## 4. Discussion

Malignant swelling can emerge as a fatal complication of space-occupying cerebellar infarction. A recently published retrospective multicenter study showed that decompressive surgery is beneficial in patients with larger cerebellar infarct volumes [5].

Overall, there is only limited literature evaluating the functional outcomes and complications of this procedure. In this retrospective single-center study, we determined a 90-day mortality rate of 27.6% for 58 patients after decompressive surgery for malignant space-occupying cerebellar infarction. This mortality rate is relatively similar to those calculated in the largest single-center studies on this subject [6,7,8]. Pfefferkorn et al. reported a 6-month mortality rate of 28% in a cohort of 57 patients [6]. While Jüttler et al. measured a mortality rate at discharge of 25% in 56 evaluated patients [7], Tsitsopoulos et al. stated a mortality rate of 31% after long-term follow-up over five years in a cohort of 32 patients [8].

We used poor functional outcome, defined as a modified Rankin Scale (mRS) score of 4–6 at 90 days after surgery, as the primary endpoint. This definition is valid according to results from a study that used long-term data comparing quality of life across mRS subgroups [9].

Our determined rate of 41.4% of patients who achieved good functional outcome coincides with results stated in the mentioned single-center studies [6,7,8]. Won et al. reported a favorable functional outcome of 61% at 1-year follow-up in surgically treated patients with cerebellar infarct volumes of 35 cm^3^ or greater [5].

Among clinical parameters, higher premorbid mRS and higher NIHSS scores on admission were significantly associated with a poor functional outcome.

Brainstem infarction being the most significant predictor for poor outcome in our analysis corresponds to the results of Pfefferkorn et al. [6]. Nonetheless, it is worth noting that one-seventh of all patients with brainstem infarction still achieved a good outcome. The presence of a brainstem infarction should therefore not solely determine the therapy. We suggest taking multiple individual factors into consideration in the therapy decision-making process.

Aspiration pneumonia, urinary tract infection and delirium were the most common problems during hospitalization in our study population, and are known to be the most frequent complications after acute stroke in general [10]. Unexpectedly, the occurrence of postoperative hyperactive delirium was associated with a good functional outcome. This contradicts the known negative impact of delirium on the prognosis of patients with acute stroke. Previous studies have demonstrated that patients with post-stroke delirium have a worse functional outcome and higher mortality [11,12]. We ascribe this result of our study to the effect of the so-called “survivorship bias”, an error that occurs when researchers focus on groups or cases that have passed a certain selection process. Patients in the “poor functional outcome group” were often in such a bad clinical state, i.e., dead within a few days or unconscious, that they were unable to express symptoms of delirium. Thus, delirium remained an underrepresented complication in this group. However, due to the small sample size of only eight cases of reported hyperactive delirium, we emphasize that this result must be treated with caution and warrants verification in a larger prospective study.

The main limitations of this study can be attributed to the retrospective and single-center design.

Our analyses relied on data from medical records that were not originally collected for research purposes. The relatively small cohort size of 58 patients limited the statistical power. Nonetheless, the main advantage of our study is that we analyzed homogeneous data from a real-world clinical practice setting of patients who may have been excluded or may have been underrepresented in prospective studies. Our study exhibits important clinical insights into a subject that has not yet been adequately investigated.

Overall, our study demonstrates that decompressive surgery in patients with malignant space-occupying cerebellar infarction can lead to a good functional outcome, especially in cases of low premorbid mRS and low NIHSS score on admission, as well as the absence of a brainstem infarction. While our study’s key results mainly coincide with results from previous studies, we exclusively considered all relevant clinical and radiological parameters in our study protocol, thus designing a potential blueprint for future prospective multicenter studies on this issue. Larger trials are required regarding this subject.

## Figures and Tables

**Table 1:** Comparison of demographic, clinical, radiological and therapeutic characteristics between patients with good functional outcome (mRS 0–3) and patients with poor functional outcome (mRS 4–6) at day 90, including results from the univariate logistic regression models with the dependent variable “poor functional outcome” (yes/no); *p*-values of the Wald statistic from the logistic models are given without adjusting for multiplicity.

	Total Cohort (n = 58)	Poor Functional Outcome, mRS 4–6 (n = 34)	Good Functional Outcome, mRS 0–3 (n = 24)	Odds Ratio	95% CI for OR	*p*-Value
Demographic characteristics						
Age (years), *mean (±SD)*	61.2 (±15.4)	60.3 (±17.3)	62.5 (±12.6)	0.991	(0.957, 1.026)	0.594
Female, *n (%)*	21 (36.2%)	15 (44.1%)	6 (25.0%)	2.368	(0.753, 7.446)	0.140
Premorbid status						
Premorbid mRS, *median (range)*	1 (0–4)	1 (0–4)	0 (0–2)	2.011	(1.165, 3.468)	**0.012**
Vascular risk factors, *n (%)*						
Arterial hypertension	38 (65.5%)	20 (58.8%)	18 (75.0%)	0.476	(0.151, 1.502)	0.206
Diabetes mellitus	18 (31.0%)	11 (32.4%)	7 (29.2%)	1.161	(0.373, 3.618)	0.796
Coronary heart disease	15 (25.9%)	10 (29.4%)	5 (20.8%)	1.583	(0.462, 5.421)	0.464
Hypercholesterolemia	18 (31.0%)	9 (26.5%)	9 (37.5%)	0.600	(0.195, 1.846)	0.373
Acute stroke severity index						
NIHSS on admission, *median (range)*	8 (0–38)	14 (0–38)	5 (0–34)	1.106	(1.035, 1.181)	**0.003**
Cause of infarction according to TOAST criteria, *n (%)*						
Large-artery atherosclerosis	10 (17.2%)	5 (14.7%)	5 (20.8%)	0.655	(0.167, 2.573)	0.545
Cardioembolism	22 (37.9%)	13 (38.2%)	9 (37.5%)	1.032	(0.351, 3.031)	0.955
Small-vessel occlusion	0					
Stroke of other determined etiology	11 (19.0%)	8 (23.5%)	3 (12.5%)	2.154	(0.507, 9.147)	0.298
Stroke of undetermined etiology	15 (25.9%)	8 (23.5%)	7 (29.2%)	0.747	(0.229, 2.442)	0.630
Imaging						
Infarct volume (cm^3^), *mean (±SD)*	54.0 (±14.3)	55.5 (±13.0)	51.9 (±15.9)	1.019	(0.981, 1.059)	0.331
Brainstem infarction, *n (%)*	21 (36.2%)	18 (52.9%)	3 (12.5%)	7.875	(1.972, 31.444)	**0.003**
Basilar artery occlusion	10 (17.2%)	8 (23.5%)	2 (8.3%)	3.385	(0.650, 17.628)	0.148
Vascular territory, *n (%)*						
PICA	39 (67.2%)	20 (58.8%)	19 (79.2%)	0.376	(0.113, 1.247)	0.110
SCA	4 (6.9%)	4 (11.8%)	0	*	*	0.999
AICA	0					
Multiple different territories	15 (25.9%)	10 (29.4%)	5 (20.8%)	1.583	(0.462, 5.421)	0.464
Side of the cerebellum						
Bilateral	19 (32.8%)	12 (35.3%)	7 (29.2%)	1.325	(0.429, 4.087)	0.625
Left	19 (32.8%)	10 (29.4%)	9 (37.5%)	0.694	(0.229, 2.102)	0.519
Right	20 (34.5%)	12 (35.3%)	8 (33.3%)	1.091	(0.362, 3.285)	0.877
Therapy						
Operation duration (minutes), *mean (±SD)*	124 (±44.1)	121 (±44.3)	128 (±44.4)	0.996	(0.984, 1.008)	0.528
Time between symptom onset and surgery (days), *median (range)*	2 (1–10)	2 (1–9)	2 (1–10)	0.922	(0.691, 1.231)	0.581
Time between malignant swelling and surgery (hours), *median (range)*	6 (1–34)	7 (1–34)	5 (2–17)	1.068	(0.958, 1.190)	0.234
EVD, *n (%)*	46 (79.3%)	30 (88.2%)	16 (66.7%)	3.750	(0.977, 14.391)	0.054
Intravenous tPA, *n (%)*	9 (15.5%)	4 (11.8%)	5 (20.8%)	0.507	(0.121, 2.128)	0.353
EVT, *n (%)*	9 (15.5%)	7 (20.6%)	2 (8.3%)	2.852	(0.537, 15.138)	0.219
Most common complications						
Aspiration pneumonia	22 (37.9%)	15 (44.1%)	7 (29.2%)	1.917	(0.632, 5.820)	0.251
Urinary tract infection	15 (25.9%)	10 (29.4%)	5 (20.8%)	1.583	(0.462, 5.421)	0.464
Hyperactive delirium	8 (13.8%)	1 (2.9%)	7 (29.2%)	0.074	(0.008, 0.648)	**0.019**

* Not enough data for a meaningful estimate.

**Table 2:** Multivariable logistic regression predicting poor functional outcome (mRS 4–6) at day 90.

	Odds Ratio	95% CI for OR	*p*-Value
Premorbid mRS	2.715	(1.166, 6.323)	0.021
NIHSS on admission	1.088	(1.014, 1.168)	0.019
Brainstem infarction	7.035	(1.255, 39.424)	0.027
Hyperactive delirium	0.020	(0.001, 0.623)	0.026

## Abbreviations

CT	computed tomography
EVD	external ventricular drain
EVT	endovascular treatment
MRI	magnetic resonance imaging
mRS	modified Rankin Scale
NIHSS	National Institutes of Health Stroke Scale
TOAST	Trial of Org 10172 in Acute Stroke Treatment
tPA	tissue plasminogen activator
